# Modelling of Animal Activity, Illuminance, and Noise on a Weaned Piglet Farm

**DOI:** 10.3390/ani13203257

**Published:** 2023-10-19

**Authors:** Maria D. Fernández, Roberto Besteiro, Tamara Arango, Manuel R. Rodríguez

**Affiliations:** 1 BioMODEM Research Group, Higher Polytechnic Engineering School, Terra Campus, University of Santiago de Compostela, 27002 Lugo, Spain; manuelramiro.rodriguez@usc.es; 2 Animal Production Department, Centro de Investigaciones Agrarias de Mabegondo, AGACAL, 15318 A Coruña, Spain; roberto.besteiro.doval@xunta.es; 3 Juana de Vega Foundation, 15176 A Coruña, Spain; tarango@juanadevega.org

**Keywords:** cosine model, animal activity, noise level, illuminance, pig, farm

## Abstract

**Simple Summary:**

Measuring animal activity and its evolution in real time is one way of assessing animal welfare. Passive infrared sensors are a low-cost solution used in weaned piglet farming that has correlated well with human observation. In addition, using other easily measured variables that provide data about animal activity in the building, such as illuminance or noise level, is also relevant. This paper establishes relationships between animal activity as measured by passive infrared sensors, illuminance and noise levels on a conventional weaned piglet farm. Fitting a cosine model to three harmonics of animal activity has proven effective in modelling these three variables. Noise level and illuminance are easily measured variables that can provide reliable information about animal activity by implementing only one sensor, a sonometer or a lux meter. Unlike passive infrared sensors, sonometers and lux meters are not affected by the physical barriers that divide the farm.

**Abstract:**

Measuring animal activity and its evolution in real time is useful for animal welfare assessment. In addition, illuminance and noise level are two factors that can improve our understanding of animal activity. This study aims to establish relationships between animal activity as measured by passive infrared sensors, and both illuminance and noise level on a conventional weaned piglet farm. First, regression models were applied, and then cosine models with three harmonics were developed using least squares with a Generalized Reduced Gradient Nonlinear method. Finally, all the models were validated. Linear models showed positive correlations, with values between 0.40 and 0.56. Cosine models drew clear patterns of daily animal activity, illuminance and noise level with two peaks, one in the morning and one in the afternoon, coinciding with human activity inside the building, with a preference for inactivity at night-time and around midday. Cosine model fitting revealed strong correlations, both in the measurement and validation periods, for animal activity (R = 0.97 and 0.92), illuminance (R = 0.95 and 0.91) and noise level (R = 0.99 and 0.92). The developed models could be easily implemented in animal welfare monitoring systems and could provide useful information about animal activity through continuous monitoring of illuminance or noise levels.

## 1. Introduction

The behaviour of pigs is highly influenced by the environment in the building [1], although it is also affected by other parameters, such as gas concentration, illuminance, noise, or health problems [2,3,4,5,6,7]. Animal activity is an important indicator of welfare that is especially relevant in intensive systems [8] because it is an early gauge for behavioural changes [9] that are caused by poor environmental conditions in the building or by health issues, among others [1,5,9,10,11]. Therefore, measuring animal activity can provide key data to reduce deficiencies in animal welfare, thus improving production efficiency and enabling sustainable pig farming [9]. 

The activity of pigs during their different growth stages is influenced by various factors such as breed, health condition, population density, housing system or lighting, among others. Also, the time of day in which observations are conducted is highly relevant [12]. In fact, there are substantial differences in the amount of activity of pigs between daytime and night-time, and their patterns of activity show variations depending on the time of day and the stage of growth [13]. Daily activity generally fits models with two peaks [8,12,14,15], although single-peak models have also been found during the first weeks postweaning [14] or in buildings with natural ventilation [12]. In other studies, daily activity has shown four daily peaks [13].

Unlike other environmental parameters, such as room temperature, light has not received enough attention within pig farming. Although inadequate lighting is known to have a negative impact on the welfare of many animals in captivity [2,16,17,18], knowledge of the consequences of different types of illuminance on pig housing is limited. In fact, the ideal illuminance level is not yet clear [19].

Preferences of growing pigs for different illuminances were found to depend on the activities undertaken [3,4,10]. However, a number of studies concerned with illuminance in the building have reported contradictory results in terms of the preference for light or darkness [20,21,22]. Whereas [20] suggests that piglets prefer illuminated compartments, [21] claims the opposite for young pigs, and [22] does not find any preference for illuminated or non-illuminated pens. Furthermore, it has been proven that high levels of illuminance increase tolerance to other stressors (elevated concentrations of ammonia and noise) [3], which contradicts the findings reported by [23], who found less aggressive behaviours in darker pens.

Photoperiod research has determined that an increase in photoperiod length causes an increase in feed intake [24,25]. However, no positive effects on growth performance were found for long photoperiods [26,27], which may be caused by the increase in activity that occurs when the photoperiod increases [16,26].

Using natural lighting in weaned piglet buildings has proved more beneficial than using artificial lighting [28] because it reduces competition over feed and the potential startling effects when lights are turned on or off [27]. Based on the literature review conducted, although the influence of illuminance on productivity and general animal behaviour is known [4,29], the ideal illuminance level is not clear, and further research is needed on this topic [16,19]. 

Noise level is an expression of the behaviour of the pigs and, for this reason, it conveys information regarding their current health and welfare situation [30,31]. Currently, there is a need to establish indicators of animal welfare that may be relevant for professionals, and also quick and easy to implement. A number of authors have claimed that the level of noise could be one of those indicators [32,33]. There is hardly any information in the scientific literature regarding noise levels in pig farming. Accordingly, further research is needed to determine whether noise level is a suitable variable to draw conclusions about animal welfare, although it may be one of the indicators of welfare [34]. 

Some authors have not found evidence that continuous exposure to a noise level of ~80 dB (A) has negative effects on pigs, which may be due to their quick adaptation to that noise [3]. In fact, the hypothesis of a relationship between high levels of noise and animal welfare is not confirmed, which has led to the conclusion that noise levels under 85 dB do not seem to affect animal welfare [34]. However, repeated exposure to 90 dB may cause stress to growing pigs, affecting productivity and animal welfare [35,36]. Moreover, a correct interpretation of their vocalisations may determine the causes for a lower level of welfare [37]. The vocalisations and other noises performed by the animals contain information concerning their emotional, physiological and individual state, which can prove useful in assessing their health condition and welfare [30,38], as well as the environment of the stable [39]. This may contribute to the early detection of several problems such as the outbreak of illnesses, or the prevention of escalations in aggressiveness. Various authors estimated pig welfare by analysing the sound waves they produced [33,40] and even drew a distinction for noises that were caused by pain [41,42]. The time of day was also proven to have an influence, particularly during feeding [43], with higher levels of noise detected for pigs that were fed automatically [24].

Various methods have been used to measure animal activity, among which are microphones [39,44], sensors attached to the animals [45,46], analysis of video footage [47] and other low-cost practices like passive infrared detectors (PID) [8,14]. These methods for monitoring and analysing animal behaviour are widely used to quantify levels of animal activity, albeit with the disadvantage of not allowing for individual observation of animals housed in groups. In general, these methods are used exclusively for research and require a higher level of development if they are to be used on commercial livestock farms [14] to improve health and animal welfare or to improve efficiency [48]. Passive infrared detectors have proved useful and reliable for measuring pig activity, regardless of the number of animals or the structure of the pen [11], showing strong correlations with video footage [11,14,15]. Nevertheless, passive infrared detectors pose several problems, such as placement, physical barriers on farms, area of coverage or the need to normalize data. On the other hand, even though it is technologically simple, continuous monitoring of illuminance and noise levels is not a part of current animal welfare monitoring systems. However, a deeper knowledge of the relationships between these variables and animal activity could provide an interesting, reliable and affordable tool that is easily implemented on conventional farms, and that can supply accurate and comparable data for different types of buildings. 

Illuminance and noise level are easily measured variables, which, unlike PID, are not affected by the physical barriers that divide the farm. Therefore, the aim of this study is to define linear models that relate animal activity, illuminance, and noise level, and also to model and validate the daily evolution of those variables based on cosine models that were previously designed for animal activity in weaned piglets. These models are intended to be effectively implemented on conventional farms.

## 2. Materials and Methods

### 2.1. Housing

Animal activity, illuminance and noise level were measured on a conventional farm of weaned piglets in Northwest Spain, latitude 43°0′53″, longitude 7°56′50″. Animals entered the farm from the farrowing pens after being weaned on 27 February 2017, at 21 days of age and an average weight of 7.05 kg. The weaned piglets stayed in a room for 39 days and were then transferred to the fattening farm on 6 March 2017, with an average weight of 19.23 kg. Every activity in the production process was performed by farm staff following the farm protocol, with no interventions by the research team. The selection and supervision of animals were conducted by the veterinary team of the farm. 

**Figure 1:**
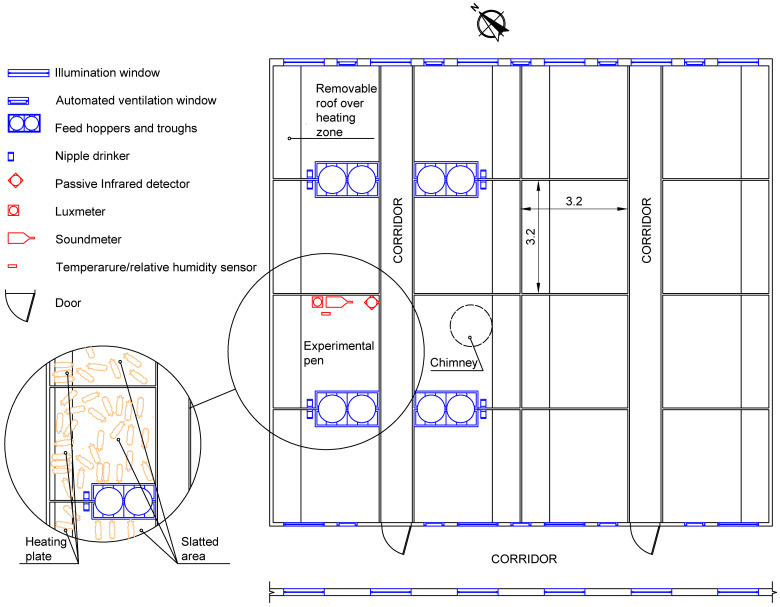
Plan of the room used for measurements.

A total of 800 piglets were housed in a central room, in order to avoid any edge effects that could affect indoor climate. The central room was a 13.00 × 14.60 m room with 16 3.20 × 3.20 m pens, with a space allowance of 0.20 m^2^, suitable for housing 5 animals by m^2^. A batch of 50 Large white × Landrace hybrid piglets was housed in the experimental pen and was used for measurements (Figure 1). The pigs were reared in accordance with EU Council Directive 2008/120/EC. EU Council Directive 2010/63/EC was not applicable, because the animals were not reared for scientific purposes, but for productive purposes, and the environment was not altered for any other reason than taking environmental measurements. Therefore, no ethical approval of the protocol was required for the present study. Piglets were fed ad libitum with compound feed provided in a double-humid hopper. The hopper was filled daily at 01:00, 08:00, 12:00, 16:00 and 20:00 h. Water was supplied through a nipple drinker placed close to the feeder. 

The pens, composed of PVC divider panels 0.70 m in height, had fully slatted floors and a 3.20 × 0.50 m heating plate, under a removable PVC roof placed at a height of 0.70 m (Figure 1). The ventilation system of the room was based on an individual exhaust fan with a chimney of 3 m in length and ten 0.58 × 0.40 m automated windows. The inner environment of the rooms was controlled by a climate control system to maintain the temperature according to the needs of the animals. During the first week after weaning, a setpoint temperature of 25 °C was assigned, which was gradually reduced and reached 22 °C when the animals left the room. In addition to natural lighting through ten 1.17 × 0.57 cm windows, artificial lightning was provided on occasion through tubular fluorescent lamps.

### 2.2. Measurements

Measurements were taken in the experimental pen over two periods. The first period of measurements (15–22 March) was used for the generation of the models and the second period (29 March–5 April) was used for the validation of the models. The average live weights of the animals, as registered at the start and at the end of each period, were: 10.14 kg on 15 March, 13.16 kg on 22 March, 15.32 kg on 29 March and 18.20 kg on 5 April. Average feed consumption was 0.49 kg/animal/day during the first period and 0.74 kg/animal/day during the second period. Between 15 and 18 March, piglets were given prestarter feed. Between 19 and 21 March, piglets were fed with a combination of prestarter and starter feeds and, from that moment, they were fed starter feed only. The studied variables were animal activity (AA), illuminance (L) and noise level (N). Furthermore, temperature (T) and humidity (RH) were measured in the animal zone, due to their relevance for animal welfare. Every variable was measured at 1 s intervals and logged at 10 min intervals, with a total of 1152 observations for each variable in each period. 

Animal activity (AA) was measured as the activation time of an infrared detector, an RX-40QZ detector (OPTEX Europe Ltd., Maidenhead, UK) with a covered area of 12 × 12 m, a covered angle of 85°, 78 detection zones and 0.125 s of minimum time of activity, according to [14]. The activation time of the sensor (s) was stored on a CR-10X datalogger (Campbell Scientific Ltd., Logan, UT, USA). The PID was placed in a corner of the weaner room away from the drinker and feeder in order to easily record the movements near the feeding equipment. In this position, and according to the covered area and angle, the PID measured activity in the whole pen. The PID was placed at 0.8 m height because of the need to cover the area under the PVC roof of the heated zone (Figure 1).

Illuminance (L, lx) was measured using a lux meter, HD 2012TAA transmitter (Delta OHM S.R.L., Caselle di Selvazzano, Italy), with a range from 0.02 to 2 klux, current output 4–20 mA, power supply 10–40 Vdc, working temperature −20–60 °C, IP66. The lux meter was placed at a height of 1.8 m in the central area of the room, above the experimental pen (Figure 1). Data were stored in a data logger HOBO-22 (Onset Computer Corporation, Bourne, MA, USA).

The noise level (N, dB) was measured with a sonometer CR–822 B (PCE Iberica S.L., Tobarra, Spain), IEC 61672-1:2003, class 2, group X, ±1.5 dB accuracy, frequency range of the microphone 5 Hz–16 kHz, ±2 dB range of measurement, broadband of 25 dB (A) at 140 dB (A), 16 Mbites of memory and 6 V DC power supply. The sonometer was placed at a height of 1.8 m in the central area of the room, above the experimental pen (Figure 1)

Temperature (T, °C) and relative humidity (RH, %) in the animal-occupied zone were measured with a temperature/relative humidity sensor, with 40–75 °C measurement range, +/−0.21 °C accuracy over the range 0–50 °C, and 0–100%, and ±2.50% accuracy over the range of 10–90% (model S-THB-M008 sensor, Onset Computer Corporation, Bourne, MA, USA). The sensor was installed in the control pen at 0.40 m height inside a metal structure that protected the equipment against aggressions from animals (Figure 1). Data were stored in a data logger HOBO-22 (Onset Computer Corporation, Bourne, MA, USA).

The divisions of the boxes limited PID measurements to the experimental pen, but measurements of illuminance and noise level were taken for all the animals in the room.

### 2.3. Data Analysis

First, a descriptive analysis of the variables was performed. Subsequently, pairwise linear models were developed for the three variables. Then, the daily variation of the variables was analysed by developing cosine models. Finally, the models were validated. Animal activity data were scaled to values between 0 and 1 to enable analysis and comparison with the values reported by other authors. The analysis was performed with Excel 2016.

#### 2.3.1. Regression Models

A pairwise regression analysis was performed between the three variables at a 95% confidence level. Such analysis provided the coefficient of multiple correlation, the coefficient of determination R^2^, the adjusted R^2^ and the standard error. Additionally, variance analysis yielded F and the critical value of F.

#### 2.3.2. Cosine Models

Data were fitted to daily evolution patterns. Patterns of animal activity with two daily peaks were used. This function is represented mathematically by an expression that has a constant part and three summands as a function of the cosine and time, according to the form [14]:

AA = A_0_ + A_1_cos(2π/24t + φ_1_) + A_2_cos(4π/24t + φ_2_) + A_3_cos(6π/24t + φ_3_) (1)

where:

A_0_ is the independent term,

t is time in hours.

A_1_, A_2_ and A_3_ are the amplitudes of the first, second and third harmonic for periods of 24 h, 12 h and 8 h, respectively.

φ_1_, φ_2_ and φ_3_ are the initial phase angles for the first, second and third harmonic, respectively.

The three variables (AA, L and N) were fitted to expression (1). Firstly, daily average data were calculated and a moving average filter with a value of 6 was used to smooth the curves. Secondly, average values were calculated every ten minutes to provide an average daily evolution pattern [49]. Thirdly, least square fitting yielded the amplitude for every harmonic A_i_ and the angles φ_i_ in expression (1) for every variable. This nonlinear problem was solved by applying one of the most popular methods to solve problems of nonlinear optimization, the Generalized Reduced Gradient (GRG) Method, requiring only that the objective function is differentiable [50]. A restriction of between 0° and 360° was used for angles and a restriction between 0 and 1 for cosine functions. 

The goodness of fit for the generation and the validation of the model was defined by the coefficient of multiple correlation, the coefficient of determination R^2^, the adjusted R^2^ and the standard error.

#### 2.3.3. Validation of the Models

Models were applied to the validation period. The validation of the model was defined by the coefficient of multiple correlation, the coefficient of determination R^2^, the adjusted R^2^ and the standard error between measured and simulated values. 

## 3. Results and Discussion

Table 1 shows statistical data for the sample composed of 1152 observations during the modelling period of the following variables: AA, scaled between the values 0 and 1, L (lx), N (dB), T (°C) and RH (%).

**Figure 2:**
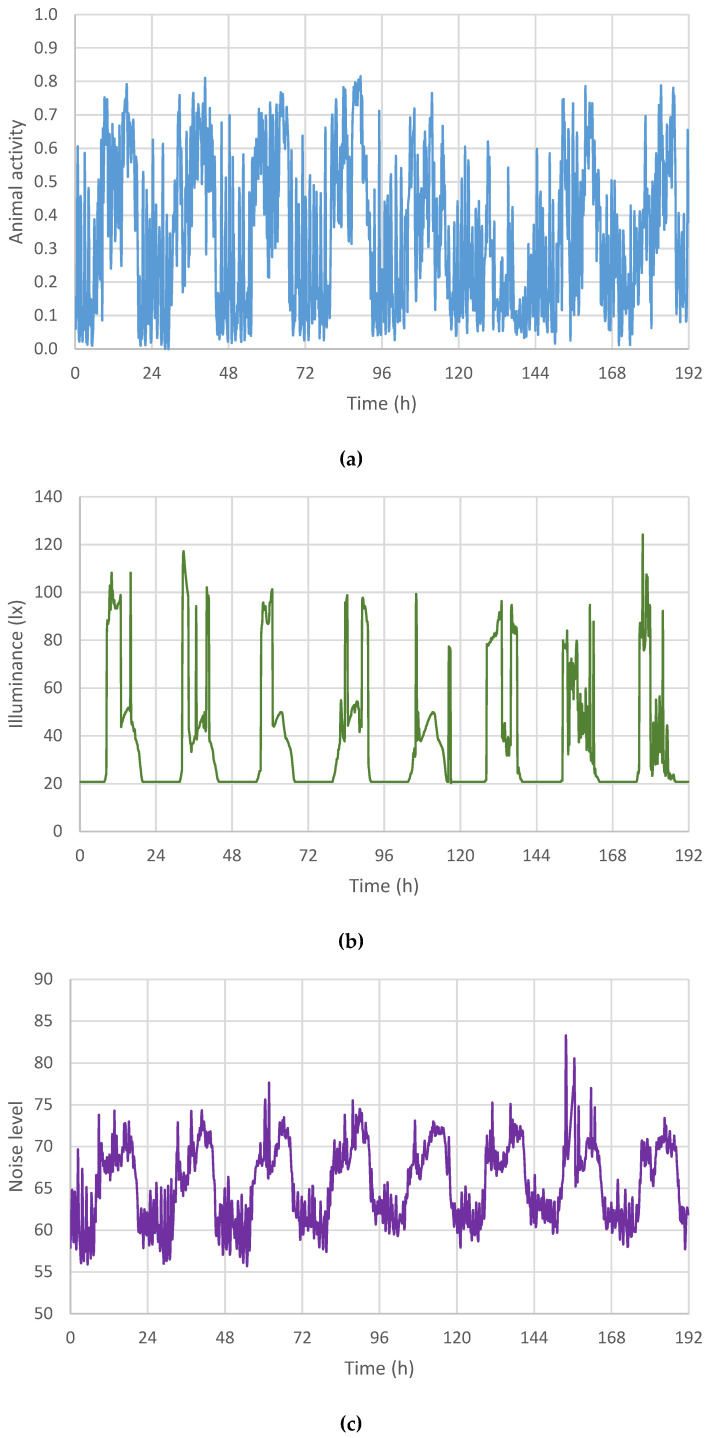
Evolution of (**a**) animal activity (AA), (**b**) illuminance (L) and (**c**) noise level (N) during the modelling period from 15 to 22 March 2017.

Animal activity is scaled to 1, but it ranges from 0.00 to 0.81 (Table 1), such that there are no 10 min intervals with continuous activity (Figure 2a). The evolution of animal activity shows lower values during the night and higher values during the day, with several daily peaks and a high oscillation between measurements. Illuminance shows a value between 20.32 and 124.22 lx. The lowest value occurs during the night and increases abruptly (Figure 2b). The level of noise is in the range between 55.70 and 83.00 dB. Temperature oscillates between 20.01 and 27.28 °C, with an average value of 25.05 °C and a standard deviation of 0.87 °C. Relative humidity shows values in the range of 38.80% to 93.60%, with an average value of 61.31% and a standard deviation of 5.67.

Illuminance values fall within the range reported in other studies [3,28]. The noise level only surpassed 73.87 dB for 4 h and 80.18 dB for 30′, which are the values established by [51] to identify that pigs are thirsty and hungry or stressed due to heat. Average values of illuminance and noise level are above 40 lx and under 85 dB (A), the values established by Council Directive 2008/120/EC of 18 December 2008. Temperature values are within the intervals set by [52], between 32 °C and 19 °C, for weaned piglets from 5 to 20 kg. The temperature exceeds the values found by [53] for 19 h and 10′ between 24 and 26 °C for the studied age. The relative humidity is stable and falls within the recommended values of 50 and 75% [54], with the exception of a period of 3 h and 10′, when the maximum value was reached. Therefore, the conditions on the farm are suitable for the variables of illuminance, noise level, temperature and relative humidity and, accordingly, for animal welfare.

The evolution of animal activity, illuminance and noise during the modelling period is shown in Figure 2.

The evolution of the variables (Figure 2) shows that there is daily periodicity. In line with most of the behaviours of growing pigs described in [12], the three studied variables show a periodic pattern. Nevertheless, the behaviour of the variables during the day shows differences, because illuminance has one daily peak, while activity and noise level have two peaks (morning and afternoon), responding to a light–dark pattern of lighting regime [2,11]. The evolution of animal activity shows lower values during the night and greater values during the day, but the large variations in measurements hinder the visualization of a clear daily pattern (Figure 2a). The daily variation of noise level shows a certain parallel with animal activity, although the wave is more stable (Figure 2a,c).

### 3.1. Regression Models 

Positive correlations were obtained between all the pairs of variables (Table 2). Despite the parallelism found between the evolution of animal activity and noise level, the strongest linear relation was found between illuminance and noise level (R = 0.50), while lower values were obtained between illuminance and animal activity (R = 0.40). In all cases, correlations were significant. This analysis confirms that the three variables are related, although linear models explain their evolution only in part due to their great complexity.

### 3.2. Cosine Models

Cosine model fitting yielded valid time patterns, according to Equation (1). Table 3 shows the coefficients and angles for each of the variables.

**Figure 3:**
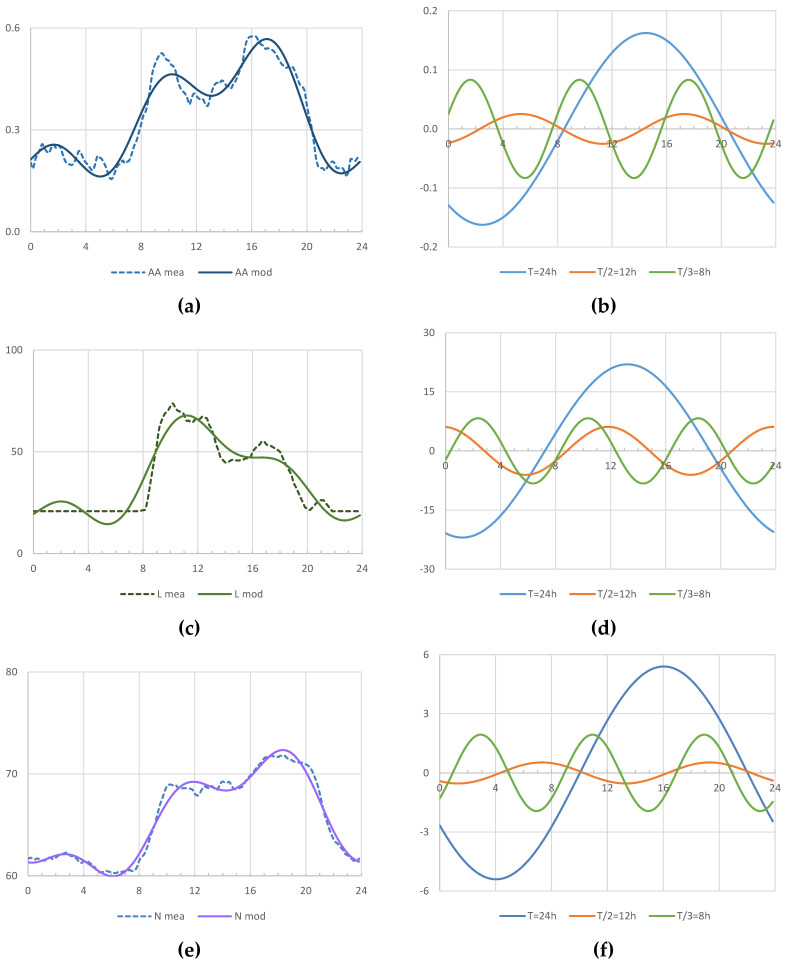
(**a**,**c**,**e**) Daily pattern measured (mea—dashed line) and modelled (mod—continuous line); (**b**,**d**,**f**) the three harmonics of the model for the variables, (**a**,**b**) animal activity (AA), (**c**,**d**) illuminance (L, lx) and (**e**,**f**) noise level (N, dB), during the modelling period.

Figure 3 shows the measured wave, the wave fitting, and the three harmonics that form the wave. Applying a cosine model draws clear patterns of daily animal activity, illuminance and noise level with two peaks, one in the morning and one in the afternoon. In the case of illuminance, the second peak is much softer.

Cosine model fitting resulted in the independent term A_0_ matching the average value. The amplitude of the first harmonic (A_1_) always had the highest value, which suggests that the light–dark pattern has a greater influence on the piglets than other variables (Table 3). The third harmonic is more relevant for every variable than the second one (A_3_ > A_2_), but amplitude is almost identical for illuminance and much higher for animal activity and noise (Figure 3b,d,f).

The analysis of the first harmonic reveals that the wave of illuminance is forward, as compared to the waves of activity and noise level, because its angle is greater (Table 3). Therefore, light can be considered as a generating factor of activity that causes a response in piglets that is delayed by 1 h 20′ for activity and 2 h 50′ for noise levels (Figure 3b,d,f). In contrast, in the second harmonic, the wave of lighting is delayed in comparison to the waves for animal activity (5 h 30′) and noise level (4 h 30′). The analysis of the third harmonic shows that illuminance is delayed by 40′ and that the noise level is delayed by 1 h 20′ as compared to animal activity. In the three harmonics, activity precedes noise level, possibly due to habits regarding feeding, water intake or games. Therefore, the time of day is essential because of the sharp differences in animal behaviour. 

Table 4 summarizes the results of the cosine model fitting to the studied variables. The low R^2^ values obtained in regression analysis improved notably when using cosine models, with high R^2^ for the three variables. Thus, cosine models can be a useful tool to study and predict these variables.

Whereas other authors presented models with up to four daily peaks [13], in this research, animal behaviour shows a pattern with two daily peaks [8,12,14,15], coinciding with human activity inside the building, with a preference for inactivity when there is less light [2,55] and around midday [12,14]. These two peaks occur after the distribution of feed (08:00 h, 12:00 h and 16:00 h), which may be the cause for such peaks, in agreement with the evolution of the third harmonic. However, there is not a similar response to the distribution of feed during night-time (01:00 h and 20:00 h). Like other models that have been developed for six different behaviours, with an approximate period of 22 h [12], the generated models can be applied to animal activity, illuminance and noise level with a fundamental period of 24 h and two harmonics (12 h and 8 h of a period, Figure 3).

In this study, conducted in conditions of natural lighting indoors, the arrival of the day involves an abrupt increase in illuminance, which is not perceived immediately by the animals, insofar as activity levels increase afterwards and in a more gradual manner (Figure 3a,c). Two levels of diurnal lighting are defined, around 80 lx in the morning and 50 lx in the afternoon (Figure 3c), when activity mostly exceeds the average value (0.3, Figure 3a). Under 40 lx, the minimum prescribed by Council Directive 2008/120/EC of 18 December 2008, activity is always below the average. After the first peak of activity during the morning, piglets are able to rest even with high values of illuminance, which is in contrast to the findings reported by [19], who found a higher level of activity for several hours in the middle of the day that were associated to a higher level of illuminance. Lighting levels in both studies were quite different (124.22 vs. 600 lx).

Noise level and illuminance are easily measured variables that could provide reliable information about animal activity by implementing a single sensor. When direct measurements of the activity of a batch housed in a room are required, PIDs and sonometers provide very similar data, but the noise level is a bit delayed in comparison to the response of the PID (between 1 h and 1 h 30′).

### 3.3. Validation of the Models

The validation of the linear model shows very similar results to the results obtained during the modelling period (Table 5).

The application of cosine models showed a good fit during the validation period, as suggested by the statistics in Table 6.

The good results obtained during the development of the model are confirmed by the validation of the model in another period, where R values range between 0.91 and 0.92 and average errors are higher than in the model development phase.

## 4. Conclusions

The following conclusions can be drawn from the analysis of the results of the experimental test, conducted in a weaner building in conditions of natural lighting indoors:

1. The linear models for the relationship between animal activity, illuminance and noise level confirm that these variables are linearly related, with correlation coefficients between 0.40 and 0.50 in the modelling period and between 0.45 and 0.56 in the validation period.
2. Applying a cosine model with three harmonics draws clear patterns of daily animal activity, illuminance and noise level with two peaks, one in the morning and one in the afternoon, coinciding with human activity inside the building, with a preference for inactivity at night and around midday. Cosine models yielded correlation values between 0.95 and 0.99 in the modelling period and between 0.91 and 0.92 in the validation period.
3. The amplitude of the first harmonic (T = 24 h) always showed the highest value, which suggests that the light–dark pattern has a greater influence on the piglets than other variables. The second (T = 12 h) and third (T = 8 h) harmonics, with shorter amplitudes, are related to daily activities of the animals, such as feeding, water intake or games.
4. Because of the simplicity of the developed models, they could be easily implemented in animal welfare monitoring systems, providing useful information about animal activity through continuous monitoring of illuminance or noise levels.

## Figures and Tables

**Table 1:** Statistical data for animal activity (AA), illuminance (L), noise level (N), temperature (T) and relative humidity (RH) during the modelling period (March 15 to March 22).

Statistic	Modelling Period				
	AA	L (lx)	N (dB)	T (°C)	RH (%)
Mean	0.343	37.122	65.559	25.052	61.305
Standard deviation	0.221	24.573	4.625	0.872	5.671
Range	0.812	103.900	27.300	7.277	54.800
Minimum	0.000	20.320	55.700	20.007	38.800
Maximum	0.812	124.220	83.000	27.284	93.600

**Table 2:** Summary of regression models and variance analysis for animal activity (AA), illuminance (L), and noise level (N). Coefficient of correlation (R), coefficient of determination (R^2^), adjusted R^2^ and standard error (SE).

Statistic	AA, L	AA, N	N, L
Intercept	0.210	−1.058	62.084
Slope	0.004	0.021	0.094
R	0.397	0.448	0.500
R^2^	0.158	0.201	0.250
Adjusted R^2^	0.157	0.200	0.249
SE	0.203	0.197	4.007
F	215.678	286.275	399.768
Critical value of F	7.020 × 10^−45^	1.822 × 10^−57^	3.134 × 10^−73^

α = 0.05.

**Table 3:** Coefficients and angles of the cosine model for animal activity (AA), illuminance (L), and noise level (N).

Amplitude and Argument	AA	L (lx)	N (dB)
A_0_	0.340	36.530	65.689
A_1_	0.162	21.969	5.400
A_2_	0.025	6.095	0.534
A_3_	0.083	8.277	1.936
φ_1_ (rad)	2.490	2.821	2.086
φ_2_ (rad)	3.505	0.092	2.453
φ_3_ (rad)	5.019	4.430	3.982

**Table 4:** Goodness of fit of the cosine model for illuminance (L), animal activity (AA) and noise level (N) during the modelling period: 15–22 March 2017. Coefficient of correlation (R), coefficient of determination (R^2^), adjusted R^2^ and standard error (SE).

Statistic	AA	L (lx)	N (dB)
R	0.971	0.952	0.991
R^2^	0.944	0.906	0.982
Adjusted R^2^	0.943	0.906	0.982
SE	0.031	5.289	0.554

α = 0.05.

**Table 5:** Results of the validation of linear models during the validation period: 29 March 2017–5 April 2017. Animal activity (AA), illuminance (L), noise level (N), coefficient of correlation (R), coefficient of determination (R^2^), adjusted R^2^ and standard error (SE).

Statistic	AA, L	AA, N	N, L
R	0.446	0.560	0.505
R^2^	0.199	0.314	0.255
Adjusted R^2^	0.198	0.313	0.255
SE	0.094	0.164	4.107

α = 0.05.

**Table 6:** Results of the validation of cosine models for animal activity (AA), illuminance (L), and noise level (N) during the validation period: 29 March 2017–5 April 2017. Coefficient of correlation (R), coefficient of determination (R^2^), adjusted R^2^ and standard error (SE).

Statistic	AA	L (lx)	N (dB)
R	0.921	0.913	0.918
R^2^	0.848	0.833	0.842
Adjusted R^2^	0.847	0.832	0.841
SE	0.051	7.051	1.630

α = 0.05.

## Data Availability

The data that support the findings of this study are available on request from the corresponding author.
